# The mediating role of health behaviors in the relationship between internal locus of control and life satisfaction in public health students

**DOI:** 10.1038/s41598-024-70178-z

**Published:** 2024-08-17

**Authors:** Paweł F. Nowak, Aleksandra M. Rogowska, Aleksandra Kwaśnicka

**Affiliations:** 1 Faculty of Physical Education and Physiotherapy, Opole University of Technology, Opole, Poland; 2 Institute of Psychology, University of Opole, Opole, Poland

**Keywords:** Life satisfaction, Mediation analysis, Multidimensional health locus of control, Health behaviors, Public health students, Public health, Quality of life

## Abstract

Well-being is a continuous process requiring decisions and actions to maintain or build health. This study examines the indirect effect of health locus of control on life satisfaction via healthy behaviors based on living systems theory. Participants were 730 students of various majors related to medicine and health, aged between 19 and 30 (*M* = 22.29, *SD* = 1.64), including 79.04% women. Self-report measures were used to assess life satisfaction, multidimensional health locus of control, and health behavior. Women scored higher than men on the total health behavior scale, especially in a healthy diet and preventive behavior. Men scored higher than women on internal health locus of control (HLC), while women scored higher than men on the powerful others HLC in making decisions about their health. The study confirmed the mediating effect of healthy behavior on the relationship between the internal HLC and life satisfaction. The present mechanism highlights the crucial role of internal motivation in increasing subjective well-being by maintaining health behaviors among young adults. The role of intervention programs focused on improving internal HLC and health behaviors is discussed.

## Introduction

Searching for ways to optimize and increase the quality of life is a significant social challenge. In many highly developed countries, trends are constantly being examined, and solutions are being sought to provide satisfactory life satisfaction. In the USA, the last decade saw a slight upward trend in life satisfaction levels along with significant optimism for the future until the outbreak of the COVID-19 pandemic^1^. In 2020, there was a decline in the life satisfaction index, and the most considerable difference between current life satisfaction and expected life satisfaction since 2008 was observed^2^. There have been declines in many mental health indicators around the world, as well as the Human Development Index^3,4^. In Poland, the level of overall life satisfaction increased successively from 1994 until 2019, after which this indicator decreased^5^. Generally, in EU countries, it was noted that a considerable decline occurred in the 18–24 age group, in which life satisfaction scores used to be among the highest and are now the lowest^6^. It is also worth noting that the pandemic has deepened the differences between the sexes. There is now a higher percentage of men who are satisfied with their lives than women, with the same level of dissatisfaction^5,6^.

### Life satisfaction in students of healthcare education

Life satisfaction is defined as a general assessment of satisfaction with one’s achievements and living conditions^7^. Satisfaction with life is one of the indicators of quality of life, which, together with indicators of mental and physical health, determine whether people develop properly^8^. We know from research that there are many concepts of achieving satisfaction in many areas, but it is difficult to isolate the factors that determine overall life satisfaction^9^. It turns out that in addition to social, financial, intellectual, emotional, personality, working and leisure, environmental, and political determinants, health conditions are also necessary, namely availability and satisfaction with various health-related services^10^.

The level of life satisfaction changes with the degree to which life needs are met, which are the source of valuable goals^11^. Young adults believe that their lives will get better and more satisfying with age, but for most, life satisfaction does not improve over time. Research indicates stabilization or even periodic declines in the assessment of their own lives^12^. Dispositional awe, mindfulness, and presence of meaning may also be important^13^. Numerous studies conducted among healthcare students in various regions of the world showed how, with the development of the COVID-19 pandemic, learning difficulties worsened, physical and mental well-being decreased, their emotional situation worsened, and life satisfaction decreased, regardless of gender^14–18^.

Public health studies prepare graduates to promote health and improve the quality of people’s lives, they are directly or indirectly prepared to work on people’s well-being, and therefore, these people should be role models, especially in their way of life and attitude towards health behaviors^19^. It is a series of responsible tasks in the service of the most valuable social values. It turns out that stress and burnout in medical and medical-related professions are large and growing problems^20,21^. Therefore, it seems essential to study the personality traits of people preparing to perform socially important professional roles, which, as is known, are correlated with subjective well-being, i.e., extraversion, self-esteem, positive affective disposition, mindfulness, optimism, the expectancy of perceived control, Pollyannaism, resilience, as well as the locus of control^9^.

### Associations between healthy lifestyle and well-being

Health behaviors are shaped during adolescence as a result of socialization and many other environmental and personal factors of the individual. Bellis et al.^22^ showed that risky behavior occurring in adulthood, as well as poor mental and physical health, are the result of unfavorable childhood experiences. It is worth noting that the period of undertaking higher education, namely entering adulthood and independence, also seems to be critical for the development of an individual lifestyle. Overall, risky behaviors occur much more often among adolescents and young adults than in other age groups^23^.

It is disturbing that during the COVID-19 pandemic, the health behaviors of nursing students, regardless of gender, have worsened^24^. Generally, medical students demonstrate a higher level of health-related behaviors than students of other fields of study^25^. It is known from many studies that gender differentiates individual health-related behaviors. Taking care of health, responsibility for one’s health, and nutrition are the domain of women, while men dominate in practices related to physical activity^26,27^. Some researchers indicate that women generally provide a more healthy lifestyle than men^28^. Longitudinal research shows that the general well-being of students has recently decreased, but men have a higher level of well-being, which is related to, among others, practicing behaviors such as getting more sleep, physical activity, greater social connections, less alcohol consumption, and less activity on social media^29^.

Satisfying needs through everyday decisions shapes an individual lifestyle. Kesebir and Diener^30^ claim that striving for happiness is one of the forms of a good life. In Western societies, diverse lifestyles are acknowledged, and they can often differ significantly. Among the younger population, healthy behaviors are influenced by numerous factors, including environmental challenges. One such recommended approach is a healthy lifestyle, which is characterized by a preponderance of behaviors that promote well-being over those that undermine it^31^. Health behaviors are any actions taken to prevent, detect disease, or improve health and well-being^32^.

The impact of healthy behaviors on life satisfaction has been the subject of extensive research. Life satisfaction, a critical cognitive component of subjective well-being, is influenced by various factors, including health behaviors^33^. In particular, regular physical activity is consistently associated with higher life satisfaction across different populations, including health professionals, older adults, and the general population^34–39^. Also, consuming a healthy diet, particularly with sufficient intake of fruits and vegetables, is linked to higher life satisfaction^34,35,38–41^. Good sleep quality is also a significant predictor of higher life satisfaction^34,39,41^. Engaging in and having an interest in daily activities was associated with higher life satisfaction among health professionals^34^. Finally, the absence of smoking was positively associated with higher life satisfaction^34,38,40,41^. Healthy lifestyle behaviors, such as healthy nutrition, stress management, regular exercise, spiritual well-being, and high-quality interpersonal relations, were related positively to life satisfaction in a sample of Turkish university students and staff^42^. However, these studies showed that healthy lifestyle behaviors and life satisfaction among university students and staff varied based on demographic variables, highlighting the need for targeted health strategies.

### Health locus of control as a factor affected healthy behavior

Health-related locus of control (HLOC) refers to an individual’s belief about the extent to which their health is controlled by internal factors (i.e., self), powerful others (e.g., doctors, physiotherapists), or chance (fate, case). People with an internal HLC believe that they can control events related to their life and health. In contrast, people with an external HLC believe that external factors, like chance or other people, determine their health and quality of life and are therefore not willing to take preventive actions^43^. Studies suggest that a higher Internal Health Locus of Control (I-HLOC) is associated with engaging in health-promoting behaviors, including physical activity, healthy diet, and adherence to medical advice^44–47^. Overall, people with an internal locus of control are more likely to eat well and exercise regularly and derive greater satisfaction from these activities^48^. Furthermore, I-HLOC is associated with better self-assessed health, physical health, mental health, and well-being^49–51^. In particular, a longitudinal study showed that a higher internal locus of control in childhood is a predictor of better health outcomes and behaviors in adulthood, including reduced risk of obesity and psychological distress^50^. The relationship between Powerful Others Health Locus of Control (P-HLOC) and health behaviors is more ambiguous and context-dependent. Some studies show that individuals who believe powerful others control their health engage in health-promoting behaviors, especially when influenced by healthcare professionals^44,47,52^. P-HLOC is particularly associated with preventive behaviors in patients with chronic diseases^52^. A high Chance Health Locus of Control (C-HLOC) is generally associated with poorer health behaviors, such as a lower likelihood of engaging in physical activity and healthy eating, as they believe their health is determined by fate^45,47,53^.

The key feature of a healthy lifestyle, a pattern of specific behaviors, is the ability to make choices. Health-related behaviors depend on beliefs regarding generalized expectations in three dimensions of locus of control^54,55^. Smoleń et al.^56^ found that university students predominantly exhibit an internal locus of health control without significant gender differences. According to Wardle et al.^57^, students from Central and Eastern European countries showed lower levels of life satisfaction than their counterparts from the Western EU. Students with low levels of life satisfaction were convicted that their health is largely determined by chance. However, an overall high level of internal locus of health control was found in students from Central and East Europe, as compared to those from West^57^. Ghorbani-Dehbalaei et al.^58^ showed that health literacy, health-related beliefs, and self-efficacy are essential factors for preventive behaviors among students. The internal health locus of control (HLC) promotes making independent decisions regarding one’s health, that is, health-promoting behaviors, regardless of the influence of other people or environmental factors. It manifests itself in individuals taking responsibility for one’s health. It is more beneficial from the point of view of public health policy^59^.

### Theoretical background of the hypothetical research model on associations between HLOC, healthy behavior, and life satisfaction

Studies suggest that an internal health locus of control is generally associated with higher life satisfaction and better mental health^51,60–68^. Popova^51^ showed that higher levels of internal locus of control are related to healthier lifestyles, which in turn contribute to higher life satisfaction in young adults (aged between 19 and 30). During the COVID-19 pandemic, individuals with a high internal locus of control were also more likely to engage in pro-environmental behaviors, which also contributed to their life satisfaction^61^. Furthermore, physical activity and social interactions were found as pathways through which an internal locus of control can enhance life satisfaction^60^. Mei et al.^68^ showed that both life satisfaction and a healthy lifestyle are positively associated with the internal HLC as well as the powerful others HLC, but chance HLC correlates negatively in a sample of Chinese students.

This study aims to examine the complex relationships between life satisfaction, health locus of control (HLC), and health behaviors among public health students, accounting for gender. Based on previous studies^51,60,61,68^ we expect that HLC and healthy behavior determine life satisfaction, and healthy behavior plays a mediating role in the relationship between HLOC and life satisfaction. Several theories can explain the expected relationships between HLOC, healthy behaviors, and life satisfaction. The eudaimonic concept of happiness assumes that life satisfaction is a cognitive component of the quality of life. Eudaimonism, as a philosophical trend, assumes involvement in the axiological sphere. The most critical factors determining well-being are self-development, the ability to change one’s life, striving to discover meaning in the actions undertaken, and fulfillment in pursuing the so-called purposeful *good* life^9,69^. Engagement in health-promoting behaviors can be considered as one of the challenges that increases self-development and leads to better physical and mental health, as well as higher well-being. It should be noted that health behaviors help to obtain the value of health, which facilitates success in life and achieving a high quality of life^70,71^.

The salutogenetic concept of health explains the relationship between health behaviors and the HLC as a process requiring decision-making and actions to maintain or build health^72^. This concept underlies contemporary health promotion, in which an essential element is emphasizing the ability to exercise control over one’s health and that of others, which is important at both individual and systemic levels of public policy^73,74^. The attribution theory^75^ assumes that HLOC serves as a crucial dynamic mechanism for motivating people to maintain healthy behaviors, which may lead to the development of life satisfaction.

The integration of all the theories mentioned above is possible from the perspective of living systems^76^. According to living systems analysis, Forrest^76^ proposed that health arises from the specific hierarchical organization of interactions between various health assets, such as energetics, restoration, mind, reproduction, and capabilities, which collectively contribute to the development of adaptation, goal attainment, needs satisfaction, and survival. Health develops throughout the lifespan due to the dynamic, non-linear interactions between individuals and their environments. The attainment of well-being for individuals is not only impacted by their health but also determined by the goals they select to pursue based on their moral values, cultural background, preferences, aspirations, and temporal context. The present study demonstrates the interaction between the mind (defined as the capacity to receive, process, and interpret data from the internal and external environments to create information that can be stored as memories and used to formulate options for action plans) and capabilities (understood as an instrumental value of health in such behavioral domains that enable individuals to move around, communicate with others, care for oneself, and interact in social situations) among university students of various public health majors, in the Polish cultural context. Based on living systems model^76^, we assume the dynamic interactions between the mind (represented in this study by HLOC and life satisfaction cognitive assessment) and capabilities (as healthy behaviors) assets that lead to better adaptation and well-being. We presume that individuals with a strong sense of health control (mind) can better manage their health-promoting behaviors (capabilities), which ultimately contribute to better self-evaluation of life satisfaction (primarily health-related) and enhanced well-being.

**Figure 1:**
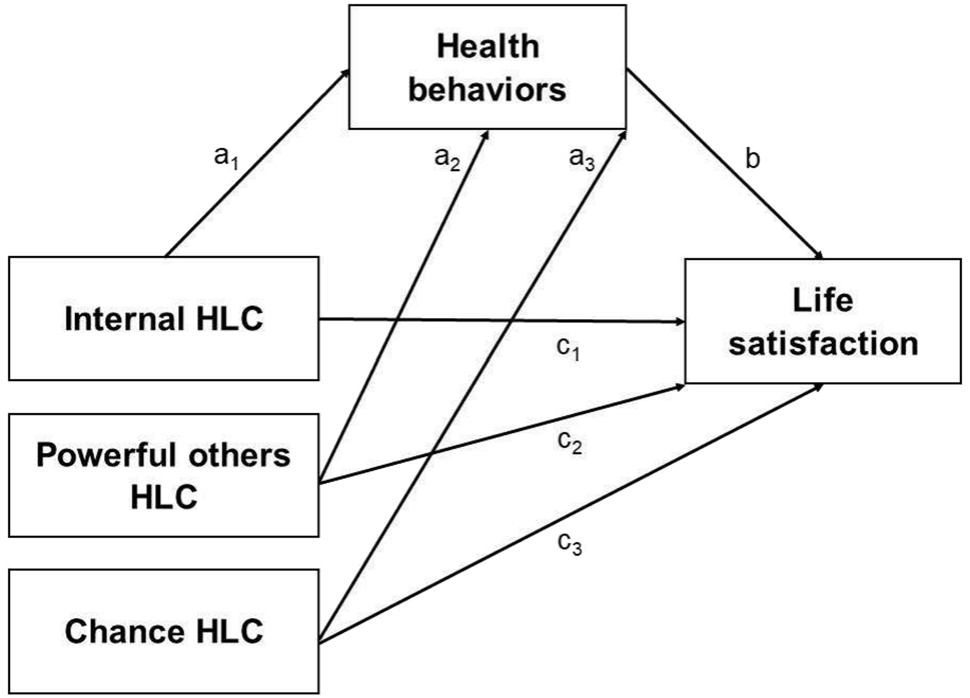
The mediating effect of health behaviors on the relationships between health locus of control (HLC) and life satisfaction (hypothetical model of mediation).

Understanding the relationships in the assumed research model of mediation is the basis for taking individual responsibility for health and life satisfaction, which is beneficial from the point of view of public health. Students of various health fields should be role models for their future patients. Therefore, it is crucial to investigate health and well-being behaviors and beliefs in the public health student population. Our previous studies showed that healthy behaviors can play a mediating role in the relationship between optimism and life satisfaction in healthcare students^77^. In particular, positive mental attitudes, a component of healthy behaviors, mediate the relationship between optimism and life satisfaction^77^. However, the mediating role of healthy behaviors was never examined in the relationship between HLOC and life satisfaction among healthcare students. Previous research^68^ has proven that a healthy lifestyle is a mediator in the relationship between HLOC in all scales (I-HLOC, P-HLOC, and C-HLOC) and life satisfaction in Chinese university students of various fields and majors. However, it is unclear whether the same associations would be observed among public health students in a different cultural context. The present study fills this gap and aims to examine the mediating effect of health behaviors in the relationship between health locus of control and life satisfaction among students of various public health majors from Poland. In the context of the previous research, the following hypotheses were formulated (Fig. 1):

Women have higher levels of health behaviors than men^28^, while men have a higher level of I-HLOC than women^78^.

Health behaviors are positively predicted by an I-HLOC (Fig. 1, path a_1_) and P-HLOC (Fig. 1, path a_2_) while negatively predicted by C-HLOC (Fig. 1, path a_3_)^44–48,50,52,53,68^.

Health behaviors are positively related to life satisfaction (Fig. 1, path b)^33–42,66,68^.

Life satisfaction is positively predicted by an internal HLC (Fig. 1, path c_1_) and powerful others HLC (Fig. 1, path c_2_) while negatively predicted by chance HLC (Fig. 1, path c_3_)^51,60–68^.

Health behaviors mediate the relationship between all three dimensions of HLC and life satisfaction^68^.

## Methods

### Sample characteristics and procedure

The cross-sectional study was performed at two large universities in Poland by using a paper-and-pencil questionnaire technique in an auditorium survey at the end of university lectures. The Research Ethics Committee approved the study protocol at the University of Opole, Poland (8/2020). The study followed the ethical requirements of anonymity and voluntariness of participation. Following the Helsinki Declaration, written informed consent was obtained from each university student before inclusion. The authors of the study received no specific funding for this work.

The research sample consists of 730 students aged between 19 and 30 (*M* = 22.29, *SD* = 1.64) from various majors related to medicine and health, like nursing, obstetrics, public health, medical rescue service, dietetics, and physiotherapy (Table 1). All participants attend the Medical University of Warsaw or the University of Rzeszów, Poland. The majority of this group are women and people with healthy body mass indexes (BMI), as determined by self-reported measurements of weight and height.

**Table 1:** Sample characteristics.

Variable	*N*	%
Gender		
Women	577	79.04
Men	153	20.96
Major		
Nursing	80	10.96
Obstetrics	52	7.12
Public health	302	41.37
Medical rescue service	77	10.55
Dietetics	121	16.58
Physical activity	98	13.42
BMI		
Underweight	66	9.07
Normal (healthy weight)	573	78.71
Overweight	78	10.71
Obese	11	1.51

### Measures

#### Sociodemographic

Demographic statistics were self-reported by participants, including age (years), gender (women, men), height (in centimeters), weight (in kilograms), and study major (nursing, obstetrics, public health, medical rescue service, dietetics, and physical activity). Body mass index (BMI) was calculated from weight and height, indicating underweight (< 18.5), healthy weight (19–24), overweight (25–29), and obese (> 30).

#### Health locus of control

The Multidimensional Health Locus of Control (MHLC) scale is designed to measure beliefs about an individual’s health agency^79,80^. The 18-item questionnaire allows for the measurement of three dimensions of the health locus of control: internal (MHCL-I; e.g., “I am in control of my health”), powerful others (MHCL-P; e.g., “Whenever I don’t feel well, I should consult a medically trained professional”), and chance (MHCL-C, e.g., “My good health is largely a matter of good fortune”). Each subscale consists of six statements that participants grade on a scale from 1 (Strongly disagree) to 6 (Strongly agree). The total score on each scale is the sum of the scores from individual items. Original Cronbach’s alfa coefficients were 0.77, 0.67, and 0.75 for each subscale, respectively^79^. The reliability of the Polish adaptation (Cronbach’s α) was as follows: 0.74 for MHLC-I, 0.54 for MHLC-P, and 0.69 for MHLC-C^59^. In the present study, the Cronbach’s alphas were 0.71, 0.68, and 0.73 for MHLC-I, MHLC-P, and MHLC-C, respectively.

#### Health behaviors

Health behaviors were assessed by the Health Behaviors Inventory (HBI) developed in the Polish language by Juczyński^59^. It consists of 25 statements describing various health-related behaviors. Participants rate the frequency of each behavior on a 5-point Likert scale (1 = Almost never, 2 = Rarely, 3 = From time to time, 4 = Often, and 5 = Nearly always). The instrument allows for the calculation of four subscales concerning different kinds of health behaviors: Healthy Diet (HD; e.g., “I eat a lot of vegetables and fruit”), Preventive Behavior (PB; e.g., “I undergo medical examinations regularly”), Positive Mental Attitudes (PMA; e.g., “I avoid situations that depress me”), and Healthy Practices (HP; e.g., “I sleep enough”). The reliability coefficient (Cronbach’s α) in the original study was 0.85 for the whole scale, and coefficients for the subscales were between 0.60 and 0.65^59^. Reliability coefficients in this study were Cronbach’s α = 0.83 for the total HBI and 0.84, 0.55, 0.70, and 0.57 for the HD, PB, PMA, and HP scales, respectively. Because the reliability of the PMA and HP scales was insufficient, only the total HBI score will be tested in further statistical analyses.

#### Satisfaction with life

The dependent variable, life satisfaction, was measured using the Satisfaction with Life Scale (SWLS) created by Diener et al.^7^. It is a short 5-item scale (e.g., “In most ways my life is close to my ideal”) with a seven-point Likert-type answer scale (ranging from 1 = Strongly disagree to 7 = Strongly agree). The total score is calculated as a sum of all five items and ranges between 5 and 35, with higher scores meaning greater life satisfaction. The reliability assessed by Cronbach’s α was 0.81 in the Polish adaptation^59^ and 0.83 in the current study.

### Statistical analyses

Descriptive statistics, like ranges, mean (*M*), standard deviation (*SD*), median (*Mdn*), skewness, and kurtosis, were performed as a preliminary analysis to examine the parametric properties of the data (Table 2). Since the sample size was large (*N* = 730) and skewness and kurtosis ranged between − 1 and + 1, a normal data distribution is considered, so parametric statistical tests were performed in the next part of the study. Gender differences were assessed using the independent samples Student’s *t*-tests, with Cohen’s *d* for an effect size assessment. Pearson’s *r*-test of correlations was performed to examine associations between variables. The mediation analysis was performed to test the indirect effect of internal health locus of control on life satisfaction among public health students via health behaviors. Gender was included in the analysis as a covariate. Bootstrapping with 5000 replications was used to increase the accuracy of results. All analyses were performed using IBM SPSS Statistics (version 26)^81^. Mediation analysis was conducted using PROCESS ver. 3.5 macro for SPSS, designed by Hayes^82,83^.

**Table 2:** Descriptive statistics (*N* = 730).

Variable (Scale)	Ranges	*M*	*SD*	*Mdn*	Skewness	Kurtosis
Health locus of control (MHLC)						
Internal HLC (MHLC-I)	8–36	26.77	4.58	27	− 0.56	0.65
Powerful others HLC (MHLC-P)	6–36	16.94	5.10	17	0.08	− 0.08
Chance HLC (MHLC-C)	6–36	17.30	5.35	17	0.18	− 0.34
Healthy behavior (HBI)	38–113	81.47	12.37	82	− 0.37	0.64
Healthy Diet (HD)	1–5	3.53	0.81	4	− 0.31	− 0.33
Preventive Behavior (PB)	1–5	3.34	0.69	3	− 0.09	− 0.23
Positive Mental Attitudes (PMA)	1–5	3.44	0.66	4	− 0.43	0.35
Healthy Practices (HP)	1–5	3.26	0.69	3	− 0.39	0.08
Life satisfaction (SWLS)	5–35	21.5	5.16	21	− 0.21	0.48

MHLC = Multidimensional Health Locus of Control, HLC = health locus of control; HBI = Health Behaviors Inventory; SWLS = Satisfaction With Life Scale.

## Results

### Descriptive statistics

Descriptive statistics for life satisfaction (SWLS), healthy behavior (HBI), and three scales of health locus of control (MHLC-I, MHLC-P, and MLHC-C) are presented in Table 2. Among the three sales of HLOC, the highest scores were presented in I-HLOC, while the lowest was in P-HLOC. Students showed the highest scores in healthy diet but the lowest in healthy practices, taking into account health behaviors. The mean score of life satisfaction indicates the respondents are slightly satisfied with their lives.

Associations between variables were examined using Pearson’s correlations (Table 3) as a preliminary analysis. Life satisfaction is positively related to health behaviors and internal HLOC. Health behaviors are related positively to both I-HLOC and P-HLOC but negatively to C-HLOC. Among HLOC scales, I-HLOC is associated positively with P-HLOC, and P-HLOC is related positively to C-HLOC.

**Table 3:** Pearson’s *r* correlations between measured variables (*N* = 730).

Variable	1	2	3	4	5	6	7	8
1. Satisfaction with life								
2. Health behaviors	0.41***							
3. Healthy diet	0.19***	0.73***						
4. Preventive behavior	0.25***	0.72***	0.38***					
5. Positive mental attitudes	0.53***	0.72***	0.31***	0.39***				
6. healthy practices	0.24***	0.72***	0.35***	0.32***	0.45***			
Health locus of control (HLC)								
7. Internal HLC	0.10**	0.27***	0.17***	0.23***	0.24***	0.14***		
8. Powerful others HLC	0.07	0.17***	0.04	0.21***	0.12**	0.15***	0.08*	
9. Chance HLC	− 0.05	− 0.09*	− 0.09*	− 0.11**	− 0.5	0.01	− 0.07	0.33***

*p < 0.05, **p < 0.01, ***p < 0.001.

Life satisfaction, healthy behavior, and three scales of health locus of control were compared across men and women, using independent samples Student’s *t*-test (Table 4), to verify hypothesis H1. Women are engaged in health behaviors significantly more often than men. In particular, women outperform men in a healthy diet and preventive behavior. When it comes to the HLOC, women scored higher than men in P-HLOC, while men scored higher in I-HLOC than women. However, the effect size for all gender differences was small (Table 3).

**Table 4:** Independent samples Student’s *t*-test for gender comparison in life satisfaction, healthy behavior, and health locus of control (*N* = 730).

Variable	Men (*n* = 153)		Women (*n* = 577)		*t*(728)	*p*	*d*
	*M*	*SD*	*M*	*SD*			
Life satisfaction	21.57	5.07	21.48	5.19	0.19	0.848	0.02
Health behaviors	77.51	13.26	82.53	11.92	− 4.52	< 0.001	− 0.41
Healthy diet	3.12	0.83	3.64	0.77	− 7.31	< 0.001	− 0.66
Preventive behavior	3.14	0.72	3.40	0.67	− 4.05	< 0.001	− 0.38
Positive mental attitudes	3.47	0.71	3.44	0.65	0.44	0.658	0.04
Healthy practices	3.20	0.69	3.28	0.69	− 1.29	0.199	− 0.12
Health locus of control (HLC)							
Internal HLC	27.67	4.51	26.53	4.58	2.75	0.006	0.25
Powerful others HLC	16.07	5.15	17.17	5.07	− 2.36	0.018	− 0.22
Chance HLC	17.12	5.65	17.35	5.27	− 0.47	0.641	− 0.04

### Mediation analysis

**Figure 2:**
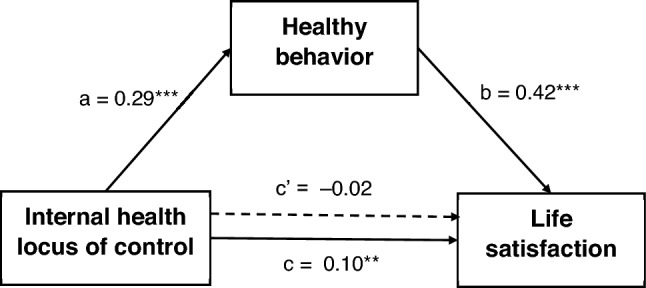
A path model for the mediating effect of healthy behaviors on the relationship between internal health locus of control and life satisfaction (*N* = 730). The numbers in the figure represent standardized regression coefficients (**β**). **p < 0.01, ***p < 0.001.

A mediation analysis assumes that the total effect is significant. Since powerful others HLC and chance HLC were unrelated to life satisfaction (Table 3), the mediation analysis was not conducted for these two variables (the total effect was insignificant). Therefore, the mediation analysis was performed only once to examine the mediating effect of health behaviors on the relationship between internal HLC and life satisfaction among healthcare students. Gender was included in the mediation model as a confounder variable. The results are shown in Table 5 and Fig. 2.

**Table 5:** Mediation analysis for the indirect effect of internal health locus of control on life satisfaction via health behaviors.

				Boot 95% CI				
Type	Effect	*b*	*SE b*	Lower	Upper	β	*t*	*p*
Indirect	Internal HLC ⇒ HBI ⇒ SWLS	0.14	0.02	0.09	0.18	0.12	4.26	< 0.001
Component	Internal HLC ⇒ HBI	0.75	0.10	0.59	0.96	0.29	8.15	< 0.001
	HBI ⇒ SWLS	0.18	0.02	0.15	0.21	0.42	11.81	< 0.001
Direct	Internal HLC ⇒ SWLS	− 0.02	0.04	− 0.10	0.06	− 0.02	− 0.49	0.625
Total	Internal HLC ⇒ SWLS	0.12	0.04	0.97	0.19	0.10	2.80	0.005

HLC = health locus of control, HBI = Health Behaviors Inventory, SWLS = Satisfaction With Life Scale, CI = confidence interval.

The total effect of internal HLC on life satisfaction was significant, but gender was not related to this association, *R*^2^ = 0.01, *F*(2, 727) = 3.95, *p* = 0.020. I-HLOC (**β** = 0.29, *p* < 0.001) and male gender (**β** =  − 0.19, *p* < 0.001) were significant predictors of health behaviors, *R*^2^ = 0.11, *F*(2, 727) = 44.32, *p* < 0.001. Also, health behaviors (**β** = 0.42, *p* < 0.001) and female gender (**β** = 0.08, *p* = 0.024) were predictors of life satisfaction, *R*^2^ = 0.17, *F*(3, 726) = 49.63, *p* < 0.001. When a mediator (health behaviors) was included in the analysis, the association between I-HLOC and life satisfaction decreased almost to zero. It was insignificant, indicating that healthy behavior fully mediates the relationship between I-HLOC and life satisfaction.

## Discussion

The study revealed that gender plays a role in distinguishing the participants’ health behaviors and health locus of control. In general, women tend to exhibit greater levels of health behaviors. However, it is crucial to consider the variations among different categories of such behaviors. Women exhibit distinctive patterns in their nutritional habits and proactive measures, with cultural factors being considered the primary driver of these differences. A woman is typically expected to manage the organization of family life. One way in which she fulfills this responsibility is by displaying concern for the well-being of her family members through actions such as preparing meals and engaging in preventive behavior, including arranging for medical check-ups. Furthermore, the role of motherhood often motivates women to prioritize their health and wellness^84,85^ Previous research indicates that men typically engage in higher levels of physical activity than women^86^. Consequently, each gender constructs its lifestyle differently.

Our investigation further verifies that men possess greater internal control over their health, while women rather believe their health depends on healthcare experts. According to a study conducted by researchers from the same cultural background, the health locus of control appears to be unrelated to gender^78^. Fiszer and Sobów’s^87^ research indicates that students attending medical universities (primarily women) scored higher in an external locus of health control than students of other universities. Overall, men outperform women in an internal HLC. It is conceivable that individuals who engage in deliberate learning may devote all available resources to their well-being. Nevertheless, this is not always the case, as functioning within a medical and paramedical environment may foster a biomedical approach to health, which involves extensive reliance on the healthcare system and thus limits individual accountability for one’s health. Shin and Lee^88^ reported a correlation between a lack of internal locus of control in unmarried women in their 20 s and an increased incidence of mental health problems. Researchers recommend that young individuals cultivate internal health control in their daily lives. By managing mental health challenges, individuals can enhance their life satisfaction.

This study showed that high levels of healthy behaviors are associated with increased life satisfaction in the group of public health students. The present findings confirm previous studies performed in various subgroups and populations^33–42,66,68^. It should be emphasized that young adults often exhibit behaviors that may not be conducive to good health (e.g., smoking, excessive consumption of alcohol or psychoactive substances, and engaging in unhealthy lifestyle habits). The pursuit of effective methods for fostering healthy behaviors has been a longstanding endeavor in Western societies, as research unequivocally demonstrates that modifications in health practices result in enhanced subjective well-being^33,89^. Life satisfaction is positively associated with various health-promoting behaviors such as abstaining from smoking, engaging in regular physical activity, utilizing sun protection, consuming fruits, and limiting fat intake^38,90^. The longitudinal study evidenced that health-promoting behaviors are a predictor of subjective well-being^91^. People who are characterized by a high level of life satisfaction are more likely to perceive their lives as pleasant, valuable, and meaningful.

Health behaviors are determined by the culture in which a person lives. Health competencies obviously play a significant role in maintaining a healthy lifestyle, prevention, and health promotion, but they are also significantly related to life satisfaction^92^. It, therefore, seems that systemic educational activities in the above area, undertaken during studies at the university, should constitute a standard element of education, as they can significantly increase the resources of broadly understood well-being necessary for effective functioning. Grace-Farfaglia et al.^93^ indicate that practicing a healthy lifestyle affects life satisfaction, especially in men. Our research shows that it is worth developing awareness of the individual impact on one’s health and creating positive attitudes towards health and working on it.

In accordance with other research^51,60–68^, the present study has revealed a positive association between life satisfaction and the internal health locus of control. However, P-HLOC and C-HLOC were unrelated to life satisfaction in this study. Previous studies showed inconsistency in the relationship between external HLOC and well-being. For example, the belief in chance and powerful others was unrelated to life satisfaction among Polish residents of veteran homes for the elderly^62^, which seems in line with this study. In contrast, Mei^68^ found a positive relationship of life satisfaction with both I-HLOC and P-HLOC and a negative association with C-HLOC among Chinese university students. The belief that powerful others controlling health may negatively affect life satisfaction among both older adults and those with chronic conditions^66,67^. Aso, Abu-Bader et al.^94^ proved that locus of control, as well as perceived physical health, social support, and emotional balance, are significant predictors of life satisfaction among older people. However, in older adults, the relationship between health locus of control and life satisfaction was influenced by factors such as health status and income, but internal control remains a significant predictor of life satisfaction independent of these factors^64^. The studies mentioned above and our findings collectively suggest that I-HLOC can play a crucial role in well-being formation.

The internal HLC, as an element determining life satisfaction, is associated with having control over and managing one’s life. The best area to train life management skills is free time. In general, the use of free time, an area that young people particularly like to decide about, is positively correlated with higher life satisfaction, especially in the case of rare forms of recreation and unusual experiences^95^. The leisure time area is usually the one that fully depends on our decisions and has consequences for maintaining health. Research shows that leisure satisfaction increases the level of overall life satisfaction^96^. As Busseri and Samani^12^ claim, much depends on the individual set of beliefs, which change across age and experience and are also subject to change during the period of education. Some researchers argue that students are a special group, and it is worth focusing on interventions that increase the level of health behaviors. The stage of life they are in is the most suitable for promoting a healthy lifestyle^97^. Therefore, it seems crucial to invest in social skills training and provide students with opportunities to play leadership roles in various organizations, clubs, and associations. It is also essential that at each level of studies, academic teachers use activating methods and various forms of learning, thanks to which students can experience taking responsibility for their development process. Special events and dedicated health programs that promote healthy behaviors and strengthen internal HLC may also be helpful^98^. Public health study programs should include subjects related to health education, health promotion, and health training.

The current research demonstrates that the existing theoretical framework within the living systems perspective^76^ can account for the process of enhancing life satisfaction through healthy behaviors among individuals with an internal health locus of control. In line with previous studies,^51,60,61,68^, the current study proved that health behaviors may play a mediator role in the relationship between internal health locus of control and life satisfaction. However, for the first time, these associations were examined in a sample of Polish students who were majoring in healthcare. In a similar study, Mei et al.^68^ showed that all three dimensions of HLC are significant predictors of both health behaviors and life satisfaction. It is important to note that there are notable differences in the research settings in the previous^68^ and our studies, including various countries, traditions, and cultures (e.g., China with Confucianism and Poland with European culture), affecting lifestyle. Moreover, students from China were involved in various academic fields^68^, whereas participants in the current study were enrolled in fields related to healthcare. The current study’s findings emphasize the significance of exercising internal control over one’s health and adopting healthy lifestyle habits, which support the majority of previous findings^51,60,61,68^. These behaviors can lead to greater enjoyment of life. Contemporary notions of health prioritize fostering the capacity for action and adaptability. Health is established through the dynamic interactions between individuals and their environment^76^. The study adds to the existing literature support for the living systems theory^76^, showing the specific interactions between mind assets concerning the sense of HLOC and capabilities assets in the healthy behaviors dimensions, which can increase the subjective sense of well-being regarding life satisfaction.

### Limitations of the study

This study may have some limitations related to the application of the results to the general population. The research was conducted in a specific region of Central and Eastern Europe. The research sample comes from two large universities in the eastern part of Poland, so the results obtained can be applied with some caution to the national student population. Cultural diversity of health behaviors and lifestyle determinants should be taken into account, even in specific regions of a given country. Given the cross-sectional design of this study, causality should be treated with caution. It would certainly be worth conducting longitudinal studies taking into account the cultural and socioeconomic diversity of European regions. In this type of research, there is also a problem with accurately capturing many interrelated health behaviors that make up lifestyle. The present study is based on self-report measures. Future studies should replicate the present study using a research tool that comprehensively and more objectively measures a representative set of health behaviors.

## Conclusions

Pro-health behaviors should be promoted among young adults, especially in men, as constructive ways to increase quality of life. This study confirmed the existence of a mechanism through which an internal sense of control over health influences life satisfaction through health-related behaviors in a sample of Polish university students of various healthcare majors. The findings support the living systems theory, adding to existing knowledge a piece of novel evidence. It is particularly important to strengthen internal HLC, especially in women, through appropriate social skills training. Systemic education in the public health area seems necessary, shaping specific beliefs about health and taking responsibility for them because health behaviors are an essential mediator in the relationship between the internal HLC and life satisfaction.

## Data Availability

The datasets generated during and/or analyzed during the current study are available from the corresponding author upon reasonable request.

## References

[1] Riley, C. *et al.* Trends and geographical variation in population thriving, struggling and suffering across the USA, 2008–2017: A retrospective repeated cross-sectional study. *BMJ Open* **11**, e043375 (2021). 34261676 10.1136/bmjopen-2020-043375 PMC8281074

[2] Riley, C. *et al.* Trends and variation in the gap between current and anticipated life satisfaction in the United States, 2008–2020. *Am. J. Public Health* **112**, 509–517 (2022). 35196041 10.2105/AJPH.2021.306589 PMC8887183

[3] WHO. *World Health Statistics 2023: Monitoring Health for the SDGs, Sustainable Development Goals*. https://www.who.int/publications/book-orders. (2023).

[4] Conceição, P. *Human Development Report 2023/2024. Breaking the Gridlock: Reimagining Cooperation in a Polarized World*. (2024).

[5] Pankowski, K. *Zadowolenie z Życia w Roku 2022. Komunikat z Badań Nr 5/2023*. http://www.cbos.pl (2023).

[6] WHO Team. *The European Health Report 2021. Taking Stock of the Health-Related Sustainable Development Goals in the COVID-19 Era with a Focus on Leaving No One Behind*. (2022).

[7] Diener, E., Emmons, R. A., Larsem, R. J. & Griffin, S. The satisfaction with life scale. *J. Pers. Assess.* **49**, 71–75 (1985). 16367493 10.1207/s15327752jpa4901_13

[8] Veenhoven, R. The study of life satisfaction. In *A comparative study of satisfaction with life in Europe* (eds Saris, W. E. *et al.*) 11–48 (Eötvös University Press, 1996).

[9] Sirgy, M. J. *The psychology of quality of life: Well-being and positive mental health* Vol. 83 (Springer International Publishing, 2021).

[10] Veenhoven, R. The four qualities of life. *J. Happiness Stud.* **2000**(1), 1–39 (2000). 10.1023/A:1010072010360

[11] Howell, R. T., Chenot, D., Hill, G. & Howell, C. J. Momentary happiness: The role of psychological need satisfaction. *J. Happiness Stud.* **12**, 1–15 (2011). 10.1007/s10902-009-9166-1

[12] Busseri, M. A. & Samani, M. N. Lay theories for life satisfaction and the belief that life gets better and better. *J. Happiness Stud.* **20**, 1647–1672 (2019). 10.1007/s10902-018-0016-x

[13] Dong, X. & Geng, L. The role of mindfulness and meaning in life in adolescents’ dispositional awe and life satisfaction: The broaden-and-build theory perspective. *Curr. Psychol.* **42**, 28911–28924 (2022). 10.1007/s12144-022-03924-z

[14] Labrague, L. J. Resilience as a mediator in the relationship between stress-associated with the Covid-19 pandemic, life satisfaction, and psychological well-being in student nurses: A cross-sectional study. *Nurse Educ. Pract.* **56**, 103182 (2021). 34508944 10.1016/j.nepr.2021.103182 PMC8425956

[15] Almhdawi, K. A. *et al.* Healthcare students’ mental and physical well-being during the COVID-19 lockdown and distance learning. *Work* **70**, 3–10 (2021). 34487002 10.3233/WOR-205309

[16] Kochan, I., Lewczuk, J. & Walczak, A. Difficulties in the functioning of “emerging adult” university students in Poland during the COVID-19 pandemic. *Colloquium Pedagogika* **48**, 47–63 (2022).

[17] Tekir, Ö. The relationship between fear of COVID-19, psychological well-being and life satisfaction in nursing students: A cross-sectional study. *PLoS One* **17**, e0264970 (2022). 35271645 10.1371/journal.pone.0264970 PMC8912239

[18] Kako, J. *et al.* Nursing students’ fear of COVID-19 and changes in life satisfaction. *Asia Pac. J. Public Health* **34**, 719–722 (2022). 35766250 10.1177/10105395221108601

[19] Fenton, K. A. Leadership in public health. In *Oxford textbook of global public health* (eds Detels, R. *et al.*) 243–260 (Oxford University Press, 2021).

[20] Reith, T. P. Burnout in United States healthcare professionals: A narrative review. *Cureus* **10**, e3681 (2018). 30761233 10.7759/cureus.3681 PMC6367114

[21] Ghahramani, S. *et al.* A systematic review and meta-analysis of burnout among healthcare workers during COVID-19. *Front. Psychiatr.* **12**, 758849 (2021). 10.3389/fpsyt.2021.758849 PMC8631719 34858231

[22] Bellis, M. A. *et al.* Does continuous trusted adult support in childhood impart life-course resilience against adverse childhood experiences - a retrospective study on adult health-harming behaviours and mental well-being. *BMC Psychiatr.* **17**, 110 (2017). 10.1186/s12888-017-1260-z PMC5364707 28335746

[23] Botsis, A. High risk behaviours in young adults: Is there a common substrate?. *Ann. Gener. Hosp. Psychiatr.* **2**, 1–14 (2003).

[24] Kupcewicz, E. *et al.* Health behaviours among nursing students in Poland during the COVID-19 pandemic. *Nutrients* **14**, 2638 (2022). 35807819 10.3390/nu14132638 PMC9268667

[25] Stasiak-Maćkowska, M. Comparison of health behaviors of medical and non-medical students: A review of the Polish literature. *Hygeia Public Health* **55**, 77–83 (2020).

[26] Gore, M. N., Yeravdekar, R. C. & Menon, K. The impact of on-campus health promotion activities on healthy lifestyle behaviours of Indian University students. *Asia Pac. J. Health Manag.* **18**, 1473 (2023).

[27] Haq, O. U., Sabghatullah, H., Ronis, K. A. & Khan, H. Assessment of health promoting lifestyle behaviors of students at the University of Malakand, Khyber Pakhtunkhwa Pakistan: A quantitative approach. *Pak. J. Public Health* **10**, 179–184 (2020). 10.32413/pjph.v10i3.502

[28] Ferreira, M. M. S. V. *et al.* University students’ lifestyles: Contributions to health promotion. *Revista de Enfermagem Referencia* **6**, 1–9 (2023).

[29] Lemyre, A., Palmer-Cooper, E. & Messina, J. P. Well-being among university students during the COVID-19 pandemic: A systematic review of longitudinal studies. *Public Health* **222**, 125–133 (2023). 37542997 10.1016/j.puhe.2023.07.001

[30] Kesebir, P. & Diener, E. In pursuit of happiness: Empirical answers to philosophical questions. Perspectives on psychological science. *Soc. Indic. Res. Ser.* **3**, 117–125 (2008). 10.1111/j.1745-6916.2008.00069.x 26158878

[31] Sharma, M. *Theoretical foundations of health education and health promotion* (Jones & Bartlett Learning, 2022).

[32] Conner, M. & Norman, P. *Predicting health behaviour : Research and practice with social cognition models* (Open University Press, 2005).

[33] Kushlev, K., Drummond, D. M. & Diener, E. Subjective well-being and health behaviors in 2.5 million Americans. *Appl. Psychol. Health Well Being* **12**(1), 166–187. 10.1111/aphw.12178 (2019). 31482675 10.1111/aphw.12178

[34] Durand-Sanchez, E. *et al.* Sociodemographic aspects and healthy behaviors associated with perceived life satisfaction in health professionals. *J. Prim. Care Commun. Health* **14**, 21501319221148332 (2023). 10.1177/21501319221148332 PMC9943957 36760092

[35] Phulkerd, S., Thapsuwan, S., Chamratrithirong, A. & Gray, R. Influence of healthy lifestyle behaviors on life satisfaction in the aging population of Thailand: A national population-based survey. *BMC Public Health* **21**, 1–10 (2021). 33407252 10.1186/s12889-020-10032-9 PMC7789197

[36] Stenlund, S. *et al.* Health behavior of working-aged Finns predicts self-reported life satisfaction in a population-based 9-years follow-up. *BMC Public Health* **21**, 1–9 (2021). 34625042 10.1186/s12889-021-11796-4 PMC8501556

[37] Martín-María, N. *et al.* Relationship between subjective well-being and healthy lifestyle behaviours in older adults: A longitudinal study. *Aging Ment. Health* **24**, 611–619 (2018). 30590962 10.1080/13607863.2018.1548567

[38] Grant, N., Wardle, J. & Steptoe, A. The relationship between life satisfaction and health behavior: A cross-cultural analysis of young adults. *Int. J. Behav. Med.* **16**, 259–268 (2009). 19319695 10.1007/s12529-009-9032-x

[39] Mateos-Lardiés, A. M. *et al.* Relationship between healthy lifestyle behaviours and subjective well-being: An european observational study. *Rev. Esp Salud Publica* **96**, e202210078–e202210078 (2022). 36263753

[40] Shi, Y., Joyce, C., Wall, R., Orpana, H. & Bancej, C. A life satisfaction approach to valuing the impact of health behaviours on subjective well-being. *BMC Public Health* **19**, 1–11 (2019). 31752788 10.1186/s12889-019-7896-5 PMC6873400

[41] Lin, M., Chen, P.-H., Chiu, W.-N. & Chen, M.-Y. Evidence of specific healthy behaviors positively associated with general life satisfaction among rural adults. *Open J. Prev. Med.* **06**, 161–169 (2016). 10.4236/ojpm.2015.66015

[42] Ergen, A. Understanding the healthy lifestyle behaviors and life satisfaction of students and staff in a university. (2016) 10.20472/IAC.2016.023.034

[43] Rotter, J. B. Generalized expectancies for internal versus external control of reinforcement. *Psychol. Monogr.* **80**, 1–28 (1966). 5340840 10.1037/h0092976

[44] Cheng, C., Cheung, M. W. L. & Lo, B. C. Y. Relationship of health locus of control with specific health behaviours and global health appraisal: A meta-analysis and effects of moderators. *Health Psychol. Rev.* **10**, 460–477 (2016). 27556686 10.1080/17437199.2016.1219672 PMC5214986

[45] Steptoe, A. & Wardle, J. Locus of control and health behaviour revisited: A multivariate analysis of young adults from 18 countries. *Br. J. Psychol.* **92**, 659–672 (2001). 11762867 10.1348/000712601162400

[46] Dogonchi, M., Mohammadzadeh, F. & Moshki, M. Investigating the relationship between health locus of control and health behaviors: A systematic review. *Open Public Health J,* **15**, (2022).

[47] Norman, P., Bennett, P., Smith, C. & Murphy, S. Health locus of control and health behaviour. *J. Health Psychol.* **3**, 171–180 (1998). 22021357 10.1177/135910539800300202

[48] Cobb-Clark, D. A., Kassenboehmer, S. C. & Schurer, S. Healthy habits: The connection between diet, exercise, and locus of control. *Health Econ. eJ.* **98**, 1–28. 10.2139/ssrn.2146274 (2012). 10.2139/ssrn.2146274

[49] Kesavayuth, D., Poyago-Theotoky, J., Tran, D. B. & Zikos, V. Locus of control, health and healthcare utilization. *Econ. Model* **86**, 227–238 (2020). 10.1016/j.econmod.2019.06.014

[50] Gale, C. R., Batty, G. D. & Deary, I. J. Locus of control at age 10 years and health outcomes and behaviors at age 30 years: The 1970 british cohort study. *Psychosom. Med.* **70**, 397–403 (2008). 18480188 10.1097/PSY.0b013e31816a719e

[51] Popova, S. Locus of control - predictor of health and subjective well – being. *Eur Med Health Pharm J* **4**, 25 (2012). 10.12955/emhpj.v4i0.367

[52] Janowski, K., Kurpas, D., Kusz, J., Mroczek, B. & Jedynak, T. Health-related behavior, profile of health locus of control and acceptance of illness in patients suffering from chronic somatic diseases. *PLoS One* **8**, e63920 (2013). 23675516 10.1371/journal.pone.0063920 PMC3651173

[53] Grisolía, J. M., Longo, A., Hutchinson, G. & Kee, F. Applying health locus of control and latent class modelling to food and physical activity choices affecting CVD risk. *Soc. Sci. Med.* **132**, 1–10 (2015). 25779694 10.1016/j.socscimed.2015.03.006

[54] Wallston, K. A. Hocus-pocus, the focus isn’t strictly on locus: Rotter’s social learning theory modified for health. *Cognit. Ther. Res.* **16**, 183–199 (1992). 10.1007/BF01173488

[55] Helmer, S. M., Krämer, A. & Mikolajczyk, R. T. Health-related locus of control and health behaviour among university students in North Rhine Westphalia Germany. *BMC Res. Notes* **5**, 1–8 (2012). 23273039 10.1186/1756-0500-5-703 PMC3544606

[56] Smoleń, E., Cipora, E., Penar-Zadarko, B. & Gazdowicz, L. Selected health behaviours presented by university students vs. health locus of control. *Med. Rev.* **4**, 274–284 (2012).

[57] Wardle, J. *et al.* Depression, perceived control, and life satisfaction in university students from Central-Eastern and Western Europe. *Int. J. Behav. Med.* **11**, 27–36 (2004). 15194517 10.1207/s15327558ijbm1101_4

[58] Ghorbani-Dehbalaei, M., Loripoor, M. & Nasirzadeh, M. The role of health beliefs and health literacy in women’s health promoting behaviours based on the health belief model: A descriptive study. *BMC Womens Health* **21**, 1–9 (2021). 34922505 10.1186/s12905-021-01564-2 PMC8684276

[59] Juczyński, Z. *Measurement tools in health promotion and psychology*. (Pracownia Testów Psychologicznych Polskiego Towarzystwa Psychologicznego, Warszawa, 2009).

[60] Kesavayuth, D., Tran, D. B. & Zikos, V. Locus of control and subjective well-being: Panel evidence from Australia. *PLoS One* **17**, e0272714 (2022). 36044403 10.1371/journal.pone.0272714 PMC9432765

[61] Hempel, C. & Roosen, J. The role of life satisfaction and locus of control in changing purchase intentions for organic and local food during the pandemic. *Food Qual. Prefer.* **96**, 104430 (2021). 34690446 10.1016/j.foodqual.2021.104430 PMC8522703

[62] Kostka, T. & Jachimowicz, V. Relationship of quality of life to dispositional optimism, health locus of control and self-efficacy in older subjects living in different environments. *Qual. Life Res.* **19**, 351–361 (2010). 20146007 10.1007/s11136-010-9601-0

[63] Quevedo, R. J. M. & Abella, M. C. Does locus of control influence subjective and psychological well-being?. *Pers. Individ. Dif.* **60**, S555 (2014).

[64] Mancini, J. A. Effects of health and income on control orientation and life satisfaction among aged public housing residents. *Int. J. Aging Human Dev.* **12**, 215–220 (1981). 10.2190/KDMD-U7FB-BFVY-64XT 7216526

[65] Rogowska, A., Zmaczyńska-Witek, B., Mazurkiewicz, M. & Kardasz, Z. The mediating effect of self-efficacy on the relationship between health locus of control and life satisfaction: A moderator role of movement disability. *Disabil. Health J.* **13**, 100923. 10.1016/j.dhjo.2020.100923 (2020). 32317244 10.1016/j.dhjo.2020.100923

[66] Johansson, B. *et al.* Health locus of control in late life: A study of genetic and environmental influences in twins aged 80 years and older. *Health Psychol.* **20**(1), 33–40 (2001). 11199063 10.1037/0278-6133.20.1.33

[67] Moshki, M., Tavakolizadeh, J., Shahroodi, M., Nabiansani, M. & Dehnoalian, A. Effect of health locus of control on the quality of life among hemodialysis patients. *J. Integr. Nurs.* **3**, 5–11 (2021). 10.4103/jin.jin_56_20

[68] Mei, Y., Zhang, Y., Yu, J., Tang, X. & Li, W. Healthy lifestyle mediates the association between health locus of control and life satisfaction among college students in Hubei, China: During the normalization stage of COVID-19 epidemic prevention and control. *Arch. Public Health* **81**, 136 (2023). 37488617 10.1186/s13690-023-01145-9 PMC10364408

[69] Waterman, A. S. Two conceptions of happiness: Contrasts of personal expressiveness (Eudaimonia) and hedonic enjoyment. *J. Pers. Soc. Psychol.* **64**, 678–691 (1993). 10.1037/0022-3514.64.4.678

[70] Downie, R. S., Tannahill, C. & Tannahill, A. *Health promotion: Models and values* (Oxford University Press, 2023).

[71] Williamson, D. L. & Carr, J. Health as a resource for everyday life: advancing the conceptualization. *Crit. Public Health* **19**, 107–122 (2009). 10.1080/09581590802376234

[72] Antonovsky, A. The structure and properties of the sense of coherence scale. *Soc. Sci. Med.* **36**, 725–733 (1993). 8480217 10.1016/0277-9536(93)90033-Z

[73] Rütten, A. The implementation of health promotion: A new structural perspective. *Soc. Sci. Med.* **41**, 1627–1637 (1995). 8746862 10.1016/0277-9536(95)00013-W

[74] Lindström, B. & Eriksson, M. Salutogenesis. *J. Epidemiol. Commun. Health* **1978**(59), 440–442 (2005). 10.1136/jech.2005.034777 PMC1757059 15911636

[75] Fiske, S. T. & Taylor, S. E. *Social cognition : From brains to culture* (SAGE, 2016).

[76] Forrest, C. B. A living systems perspective on health. *Med. Hypotheses* **82**, 209–214 (2014). 24368035 10.1016/j.mehy.2013.11.040 PMC3926937

[77] Rogowska, A. M., Nowak, P. F. & Kwaśnicka, A. Healthy behavior as a mediator in the relationship between optimism and life satisfaction in health sciences students: A cross-sectional study. *Psychol. Res. Behav. Manag.* **14**, 1877–1888 (2021). 34853542 10.2147/PRBM.S335187 PMC8627888

[78] Krumov, K. D. *et al.* Health locus of control in a pandemic situation: cross-cultural differences between European and Asian respondents. *Health Psychol. Rep.* **10**, 227–237 (2022). 38084278 10.5114/hpr.2022.115947 PMC10501430

[79] Wallston, K. A., Wallston, B. S. & DeVellis, R. Development of the multidimensional health locus of control (MHLC) scales. *Health Educ. Monogr.* **6**, 160–170 (1978). 689890 10.1177/109019817800600107

[80] Wallston, K. A. The validity of the multidimensional health locus of control scales. *J. Health Psychol.* **10**, 623–631 (2005). 16033784 10.1177/1359105305055304

[81] IBM. SPSS Statistics for Windows . Preprint at (2019).

[82] Hayes, A. F. *From guilford introduction to mediation, moderation, and conditional process analysis* (Guilford Press, 2022).

[83] Hayes, A. F. PROCESS: Macro for Windows. Preprint at (2019).

[84] Jones, A. S. & Frick, K. D. The roles of women’s health and education in family and societal health. *Women’s Health Issues* **20**, 231–233 (2010). 20620911 10.1016/j.whi.2010.04.003

[85] Grabowska, M. *et al. Kobieta w Rodzinie, w Pracy, w Przestrzeni Publicznej. Opinie i Diagnozy Nr 25*. (2013).

[86] WHO. *Global Status Report on Physical Activity 2022*. https://iris.who.int/handle/10665/363607 (2022). 10.1016/j.jshs.2022.12.006 PMC9923423 36528290

[87] Fiszer, K. & Sobów, T. Relation between health locus of control and depression among students of a medical college. *Medycyna Ogólna i Nauki o Zdrowiu* **19**, 294–299 (2013).

[88] Shin, S. & Lee, E. Relationships among the Internal Health Locus of Control, Mental Health Problems, and Subjective Well-Being of Adults in South Korea. *Healthcare (Basel)* **9**, 1588 (2021). 34828633 10.3390/healthcare9111588 PMC8620821

[89] Stenlund, S. *et al.* Changed health behavior improves subjective well-being and vice versa in a follow-up of 9 years. *Health Qual. Life Outcomes* **20**, 1–12 (2022). 35449057 10.1186/s12955-022-01972-4 PMC9027415

[90] Sran, S. K., Vats, P. & Wadhawan, P. Effect of exercise on life satisfaction and happiness. *Indian J. Health Well-being* **12**, 79–82 (2021).

[91] Stenlund, S. *et al.* Longitudinal stability and interrelations between health behavior and subjective well-being in a follow-up of nine years. *PLoS One* **16**, e0259280 (2021). 34714864 10.1371/journal.pone.0259280 PMC8555827

[92] Hirooka, N. *et al.* Association between health literacy and purpose in life and life satisfaction among health management specialists: A cross-sectional study. *Sci. Rep.* **12**, 1–7 (2022). 35585083 10.1038/s41598-022-11838-w PMC9117675

[93] Grace-Farfaglia, P., Pickett-Bernard, D., Gorman, A. W. & Dehpahlavan, J. Health philosophy of dietitians and its implications for life satisfaction: An exploratory study. *Behav. Sci. (Basel, Switzerland)* **7**(4), 67 (2017). 10.3390/bs7040067 PMC5746676 29048357

[94] Abu-Bader, S. H., Rogers, A. & Barusch, A. S. Predictors of life satisfaction in frail elderly. *J. Gerontol. Soc. Work* **38**, 3–17 (2003). 10.1300/J083v38n03_02

[95] Choung, Y., Pak, T. Y. & Chatterjee, S. Consumption and life satisfaction: The Korean evidence. *Int. J. Consum. Stud.* **45**, 1007–1019 (2021). 10.1111/ijcs.12620

[96] Tokay Argan, M. & Mersin, S. Life satisfaction, life quality, and leisure satisfaction in health professionals. *Perspect. Psychiatr. Care* **57**, 660–666 (2021). 33216397 10.1111/ppc.12592

[97] Plotnikoff, R. C. *et al.* Effectiveness of interventions targeting physical activity, nutrition and healthy weight for university and college students: A systematic review and meta-analysis. *Int. J. Behav. Nutr. Phys. Act.* **12**, 1–10 (2015). 25890337 10.1186/s12966-015-0203-7 PMC4393577

[98] Gilbert, G. G., Sawyer, R. G. & McNeill, E. B. *Health education: Creating strategies for school & community health* (Jones & Bartlett Publishers, 2014).

